# Time‐related variation in IgG subclass concentrations in a group of healthy Danish adults

**DOI:** 10.1002/iid3.464

**Published:** 2021-06-02

**Authors:** Kristina Fruerlund Rasmussen, Ulrik Sprogøe, Christian Nielsen, Dana‐Bar Shalom, Kristian Assing

**Affiliations:** ^1^ Department of Clinical Immunology University Hospital Odense Denmark; ^2^ Department of Oncology University Hospital Odense Denmark

**Keywords:** adult, immunoglobulin G, immunoglobulin G4‐related disease, observational study

## Abstract

**Introduction:**

Immunoglobulin G subclass measurements are important for the diagnostic work‐up of immunodeficiencies and immunoglobulin G4 (IgG4) related diseases. It is currently unknown whether a single sampling is truly representative for an individual's IgG subclass concentrations. This study aimed to investigate whether IgG and IgG subclass concentrations in healthy individuals are stable over time.

**Method:**

With a span of median 42 weeks, four samples from each of 54 (34M, 20F) healthy adult volunteers (24–66 years) were analyzed for IgG and IgG1–4 using turbidimetry. Concentrations were compared within and between individuals.

**Results:**

IgG and IgG subclass concentrations followed either a normal (IgG, IgG1, and IgG3) or log normal (IgG2 and IgG4) distribution. Immunoglobulin 4 demonstrated by far the widest range of concentrations between individuals (670‐fold: 0.004–2.68 g/L). Immunoglobulin G subclass variations within individuals were expressed as pooled standard deviations (PSD). These ranged from 0.056 (IgG4) to 0.955 g/L (IgG) and correlated with mean concentration of IgG or the particular IgG subclass. As a consequence, the relative PSDs (i.e., PSD divided by mean IgG or IgG subclass concentration) fell within a narrow range: 5.82%–10.1%. Based on these numbers, the 95%‐upper one‐sided confidence limits for intraindividual IgG and IgG subclass variation was calculated to range from 9.82% (IgG2) to 16.9% (IgG4).

**Conclusion:**

The study documents that IgG or IgG subclass concentrations within healthy individuals are very stable over at least 42 weeks. The expected variation for IgG4 concentrations at a 95% confidence level does not exceed ±16.9%.

**Abbreviations**
CI: confidence interval
IgG: immunoglobulin
PSD: pooled standard deviations
*SD*
: standard deviation

## 1 INTRODUCTION

Serum concentrations of immunoglobulin G (IgG) subclasses have been associated with various disorders and diseases. Low amounts of IgG subclass antibodies are associated with humoral immunodeficiency^1^, ^2^, ^3^, ^4^, ^5^, ^6^ while high amount of IgG subclass antibodies, especially IgG4, have been associated with systemic diseases, such as storiform fibrosis and obliterative phlebitis.^7^, ^8^, ^9^ Therefore, assessment of IgG1‐4 subclass concentrations is part of the diagnostic work‐up in rheumatological, gastrointestinal and neurological diseases or in the clinical evaluation of patients with primary or secondary immune deficiencies.^1^, ^10^, ^11^, ^12^, ^13^ More specifically, in the case of IgG4 related diseases, measurement of an individual's IgG4 concentration is used as a diagnostic guide.^14^ Determining IgG subclass concentrations is of expanding clinical relevance due to accumulating evidence of IgG subclass related diseases.^15^, ^16^, ^17^ Hence, it is of clinical relevance to establish variation in IgG subclass concentrations over time to determine how representative a single IgG subclass determination is. To the best of the authors' knowledge, intraindividual variation of serum IgG subclass concentrations has not previously been described. Further, only a few cross‐sectional studies on IgG subclass reference intervals in healthy individuals have been published.^18^, ^19^, ^20^, ^21^

It has previously been established that serum IgG antibodies are stable in frozen and thawed samples.^9^, ^22^, ^23^ Thus, it is possible to use previously collected and frozen samples for the purpose of estimating intraindividual variations of IgG subclass levels over a period of time covered by the collected samples.

The aim of this study is to investigate the biological intra‐ and interindividual variations in immunoglobulin IgG subclass concentrations—with particular focus on IgG4—in an array of frozen samples collected serially from a group of healthy adults.

## 2 MATERIALS AND METHODS

### 2.1 Study design

This study was part of a previously published prospective study which described intraindividual variation of anti‐A and/or anti‐B titers.^23^

Serum and plasma samples were collected from a cohort of 59 healthy volunteers consisting of blood donors or employees at the Department of Clinical Immunology, Odense University Hospital. Participants were more than 18 years old and Caucasians. Volunteers had given written informed consent to the use of the samples for research purposes. Exclusion criteria were those pertaining to Danish blood donors: presence of symptomatic autoimmune disease, type 1 diabetes, malignancy (previous/current), viral or parasitic disease with blood transmission, symptomatic infection, pregnancy, use of oral or injected medicine within two weeks before sampling or ongoing use of these medications.^24^ Volunteers were not financially compensated.

### 2.2 Blood sampling

A total of four serum and plasma samples from each volunteer were collected at approximately every third month covering a period of approximately ten months from May 2012 to August 2013. Sampling time of the day varied randomly. The samples were collected by venipuncture and fractionated serum and plasma was frozen in aliquots at −80°C within one hour after sampling.

### 2.3 IgG subclass analysis

Aliquots were thawed and IgG and IgG1, IgG2, IgG3, and IgG4 subclass concentrations were analyzed with turbidimetry using commercially available kits (Product Code NK004.S., NK006.S., NK007.S., LK008.S., LK009.S, The Binding Site) and a SPAPLUS analyzer (The Binding Site) in accordance with the manufacturer's instructions.^21^ The assay was validated for use on either plasma or serum samples. According to the manufacturer, the analytical range for the IgG4 assay is 0.003–3.4 g/L.^25^, ^26^

### 2.4 Data analysis

Basic data analysis was carried out using Microsoft Excel 2010 (Microsoft) by calculating mean individual IgG and IgG subclass concentrations, 95%‐distribution limits of interindividual mean IgG and IgG subclass concentrations and pooled standard deviations (*SD*) and 95%‐upper distribution limits of intraindividual IgG and IgG subclass variations. The pooled *SD* was calculated as:

$$\begin{array}{l} \mathrm{Square}\;\mathrm{root}\;(\;(\;\sum \mathrm{variance}\\ \;\left[\mathrm{across}\;\mathrm{four}\;\mathrm{measurements}\;\mathrm{in}\;\mathrm{an}\;\mathrm{individual}\right])\\ \;/n);n=\mathrm{number}\;\mathrm{of}\;\mathrm{individuals} \end{array}$$

Pooled relative SD for intraindividual subclass variation was calculated as:

$$\begin{array}{l} \mathrm{Square}\;\mathrm{root}\;(\;(\;\sum \mathrm{variance}/\mathrm{mean}\\ \;\times \;2\left[\mathrm{across}\;\mathrm{four}\;\mathrm{measurements}\;\mathrm{in}\;\mathrm{an}\;\mathrm{individual}\right])\\ \;/n);n=\mathrm{number}\;\mathrm{of}\;\mathrm{individuals} \end{array}$$

Confidence limits were calculated as:

$$\begin{array}{l} \mathrm{SD}\times t_{0.05}\mathrm{at}\;n-1\;\mathrm{degrees}\;\mathrm{of}\;\mathrm{freedom};n\\ \;=\mathrm{number}\;\mathrm{of}\;\mathrm{individuals}\\ \left(\mathrm{For}\;n=54,t_{0.05}\mathrm{at}\;n-1\;\mathrm{degrees}\;\mathrm{of}\;\mathrm{freedom}=1.675\right) \end{array}$$

For one‐sided confidence limits, *t*_0.05_ of one‐sided *t* distribution was used. Further statistical analysis and graphics was performed using GraphPad Prism 7 (GraphPad Software) or JMP 12.01 (SAS Institute Inc.). Evaluation of adherence to normal distribution of mean individual IgG or IgG subclass level between individuals was performed using Shapiro‐Wilks W test in JMP.

To distribute the number of individuals in age groups of fairly equal size, age intervals were set at 24–44 years, 45–54, 55–62, and 63–66 years. Test of differences of mean IgG and IgG subclass concentrations across these age groups was performed using analysis of variance.

Unless otherwise stated, the level of statistical significance was set at two‐tailed distribution *p* < .05 for all statistical tests.

## 3 RESULTS

### 3.1 Study population

Of the 59 eligible volunteers, 54 included participants completed the study. One female withdrew her consent. One male was excluded due to health issues and further three males were excluded as they failed to donate four samples. The characteristics of the participants are presented in Table 1. There was no difference in mean age between females and males (53.5 vs. 51.4 years respectively, *p* = .63). Median duration between sample collections was 14 weeks (range 7–29 weeks).

**Table 1:** Population characteristics (*N* = 54)

**Sex**	**Age (years)**	**Observation period (weeks)**	
Male	34 (63%)	54 (24–66)	42 (37–52)
Female	20 (37%)		

*Note:* Age and observation period are given as median (range).

**Figure 1:**
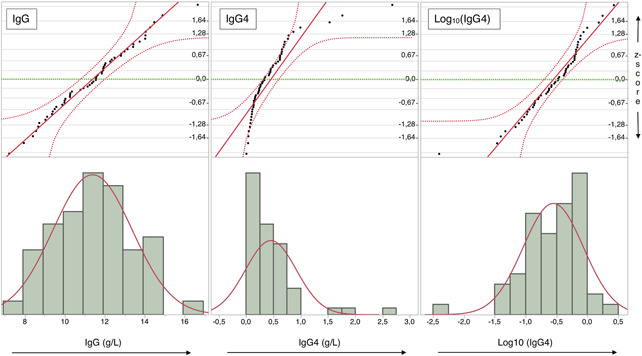
Typical distributions of IgG and IgG subclass concentrations across individuals. Normal quantile plots (top) and histograms (bottom) with fitted normal distribution (red curve) showing typical distributions of IgG and IgG4 subclass concentrations across individuals. Immunoglobulin G concentrations (Panel 1) conform to a normal distribution. For IgG4, concentrations (Panel 2 and 3) adhere to log normal distribution after removal of one outlier (Panel 3). *N* = 54 (each dot represents the mean of four measurements from an individual). IgG, immunoglobulin G

Immunoglobulin G, IgG1 and IgG3 concentrations were normally distributed (Shapiro–Wilks *p* ≥ .48) between individuals and IgG2 and IgG4 concentrations were log normally distributed (Shapiro–Wilks *p* = .097 and *p* = .50, respectively). Regarding IgG4, adhering to the log normal distribution required removal of an outlier representing the lowest IgG4 concentration measured, 0.004 g/L (all IgG4 data: Shapiro–Wilks *p* = .01). See Figure 1 for examples.

Subject to availability of serum samples, 15 out of the total 216 (6.9%) samples were EDTA‐plasma. The few plasma samples were randomly distributed across participants, with one sample from each of 11/54 (20%) participants and two samples from each of 2/54 (3.7%) participants.

The basic data on IgG and IgG1‐4 distributions across individuals (interindividual variation) are presented in Table 2. All 1080 IgG and IgG1‐4 measurements fell within the manufacturer's declared analytical ranges of the IgG and IgG‐subclass assays.

**Table 2:** Descriptive statistics for IgG and IgG subclass concentrations (g/L) (*N* = 54)

**IgG type**	**Mean (*SD*)**	**95% CI**	**Median**	**Percentiles (2.5%–97.5%)**	**Range (g/L)**
IgG	11.4 (1.98)	7.43–15.4	11.6	7.49–16.0	7.22–16.7
IgG1	6.46 (1.46)	3.52–9.40	6.44	3.79–9.98	3.76–10.6
IgG2	3.75 (1.01)	2.10–6.22^a^	3.43	1.97–5.73	1.83–5.85
IgG3	0.69 (0.28)	0.12–1.25	0.67	0.18–1.39	0.15–1.48
IgG4	0.45 (0.47)	0.03–2.67^a^	0.33	0.02–2.34	0.004–2.68

Abbreviations: CI, confidence interval; IgG, immunoglobulin G.

^a^ Estimate based on a logarithmic distribution model.

### 3.2 Intraindividual and interindividual variation

The range of IgG4 concentrations across individuals relative to those of IgG1–3 was wide (0.004–2.680 g/L). The interindividual variability of IgG4 concentrations is underscored by (Table 2) a higher ratio between IgG4 mean and IgG4 median (1.36) compared to 0.98–1.09 for IgG and IgG1–3. Data on the intraindividual variation of IgG and IgG1‐4 concentrations across four measurements (Table 3) are expressed as pooled *SD* (*SD* relating to variation over time, then pooled across participants). It is apparent that the pooled *SD* correlates to mean immunoglobulin concentration (Tables 2 and 3), indicating that variation over time for IgG or an IgG subclass is decreasing proportionally with mean concentration of that particular immunoglobulin. Pooled relative *SD*, calculated by taking into account this relationship (dividing pooled *SD* with mean of relevant IgG and IgG subclass concentrations) revealed that the differences between IgG and between the four subclasses in relative variation over time were rather small with a maximum 1.7 fold difference between the lowest (5,85%, IgG2) and the highest (10.1%, IgG4) relative *SD*. The maximum 95% relative upper CI (Table 3) of 16.9% (IgG4) indicates that IgG and IgG subclass concentrations of an individual are very stable across time

**Table 3:** Intraindividual variation in IgG and IgG subclasses across four measurements

**Ig type**	**Pooled *SD* (g/L)**	**95% Upper CI (g/L)**	**Pooled rel. *SD* (%)** a	**95% Rel. upper CI (%)** a
IgG	0.955	1.600	8.92	14.9
IgG1	0.536	0.898	8.39	14.1
IgG2	0.222	0.372	5.86	9.82
IgG3	0.058	0.097	8.40	14.1
IgG4	0.056	0.093	10.1	16.9

*Note*: With IgG1 as an example the CIs in the table may be read as: Maximum time related intraindividual variation of IgG1 at 95% probability = ±0.898 g/L. At a mean level of IgG1 of 6.46 g/L (Table 2) this corresponds to an approximate interval of IgG1 of 5.6–7.4 g/L. Alternatively, expressed relatively: IgG1 level ±14.1%, which corresponds to an approximate interval of IgG1 of 86%–114%.

Abbreviations: CI, confidence interval; IgG, immunoglobulin G.

^a^ Estimates of relative *SD* and 95% upper CI on relative variation.

**Figure 2:**
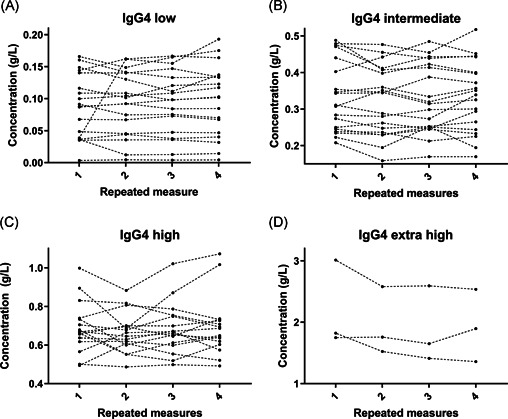
IgG4 variation over time. Time‐related variation of IgG4 concentration in 54 individuals across four measurements. Note different concentrations of Y‐axis. IgG, immunoglobulin G

For IgG4, this is further illustrated in Figure 2. Here, all IgG4 concentrations are visualized with each participant's four measurements represented by a dotted line. Only for a few subjects, there was a significant fluctuation of IgG4 level across time.

### 3.3 Variation in immunoglobulin level in relation to sex and age

No statistically significant differences in mean IgG or mean IgG1–4 subclass concentrations according to sex were identified (Student's *T* test, .10 < *p* < .68, data not shown). The four defined age groups (24–44, 45–54, 55–62, and 63–66 years) were comprised of 11 to 15 persons. There was no statistically significant association between mean IgG or IgG1–4 subclass concentrations and age group (.10 < *p* < .33, data not shown).

## 4 DISCUSSION

The main novel finding of the study is with regard to the intraindividual variations in concentrations of IgG and IgG1–4 subclasses by repeated sampling over a time span of 10 months. Overall, variations between repeated measurements were small. According to the data on pooled standard deviations, intraindividual variation range from 0.056 g/L for IgG4 to 0.955 g/L for total IgG. Interestingly, the pooled *SD* pertaining to an IgG subclass was proportional to the mean concentration level of the particular subclass. This allowed for the calculation of pooled relative standard deviations as a measure of the time‐related variations of the IgG subclasses relative to the subclass concentrations. Further calculation yielded one‐sided 95%‐CIs on the relative variations. The highest, 16.9% was seen for IgG4, but they were fairly consistent across subclasses and total IgG with no clear increasing or decreasing tendency relative to mean immunoglobulin level (Table 3). The main implication of these findings is that regardless of IgG subclass, intraindividual time‐related variation do not exceed 17%, meaning that any single measurement of IgG or IgG1–4 subclass with a 95% certainty will lie within an 83%–117% range of the “true” value of the measured total IgG or IgG subclass for that particular individual.

Compared to the concentration of total IgG and each of the other three IgG subclasses, interindividual IgG4 concentrations spanned the widest range of values. In fact, three participants demonstrated mean IgG4 values above 1.35 g/L (male 65 years, male 36 years and female 47 years), which is normally considered the upper normal limit, when considering IgG4 related disease.^7^, ^27^ As the study participants all entered the study as healthy individuals without signs of chronic disease, there is no indication that these three individuals may have had IgG4 related disease. At the other end of the IgG4 spectrum, four of 54 participants presented with mean IgG4 values less than 0.05 g/L. However, as shown by Figure 2 regardless of the mean IgG4 level of an individual the intraindividual IgG4 variation was at a most modest level. The wide range of IgG4 concentrations must therefore be assumed to be an indication of the true biological variation of IgG4 rather than caused by measurement errors.

This wide interindividual IgG4 range is consistent with previous studies on the interindividual variation in other human biological markers, for example, lymphocyte subsets.^28^, ^29^, ^30^ Similarly, small intraindividual variation has also been demonstrated in other studies, for example, cytokines or lymphocytes.^30^

There are limitations to this study. The main being that it includes a relatively small number of participants and that the age span of the participants was limited to 24–66 years. It is clear, that a larger study population with inclusion of older and younger participants would have yielded results that are more generalizable. The fact that the participants were all healthy adults further makes it difficult to draw a direct comparison of results the study to various patient groups. Although the wide range of concentration of IgG4 gives confidence on what variations to expect in patient groups with IgG4 related disease.

An additional caveat is that the study was confined to four measurements per subject with a median distance of 14 weeks between samplings. As such, it cannot be ruled out that larger IgG level variations than those presented here, may be the case when samplings are years or further apart. On the other hand, since the time interval between samplings from the individual participants carried some random variation, it is far less likely that short term IgG variations are present, but not detected by this study. We conclude that the data presented in this study demonstrates a high degree of stability in IgG subclass concentrations within adult healthy Danish adults over time. This knowledge is applicable in the clinic for investigating patients for IgG subclass related disease.

## CONFLICT OF INTERESTS

The authors declare that there are no conflict of interests.

## AUTHOR CONTRIBUTIONS

Kristina Fruerlund Rasmussen has contributed to the conception and design of work. She has analysed the data. She has drafted the work and approved the final version of the article. Ulrik Sprogøe carried out data analysis and statistical testing. Further, he cowrote and edited the manuscript. Christian Nielsen has been involved in laboratory work analyzing the data. He has drafted the work and approved the final version of the article. Dana Bar Shalom has contributed to laboratory work, co‐edited and approved the final version of the article. Kristian Assing has collected data and consents. He has contributed to the conception and design of work. He supervised the work. He has drafted the work and approved the final version of the article.

## ETHICS STATEMENT

All participants had given written and informed consent before entry into the study. The research protocol was reviewed and approved by the Regional Committees on Health Research Ethics for Southern Denmark (Protocol‐identification: S‐20110085).

## Data Availability

The data that support the findings of this study are available on request from the corresponding author. The data are not publicly available due to privacy or ethical restrictions.

## References

[1] Parker AR , Skold M , Ramsden DB , Ocejo‐Vinyals JG , Lopez‐Hoyos M , Harding S . The clinical utility of measuring IgG subclass immunoglobulins during immunological investigation for suspected primary antibody deficiencies. Lab Med. 2017;48(4):314‐325. 29126302 10.1093/labmed/lmx058 PMC5907904

[2] Soderstrom T , Soderstrom R , Avanzini A , Brandtzaeg P , Karlsson G , Hanson LA . Immunoglobulin G subclass deficiencies. Int Arch Allergy Appl Immunol. 1987;82(3‐4):476‐480. 3570516 10.1159/000234258

[3] Schatorje EJ , de Jong E , van Hout RW , Garcia Vivas Y , de Vries E . The challenge of immunoglobulin‐G subclass deficiency and specific polysaccharide antibody deficiency—a Dutch pediatric cohort study. J Clin Immunol. 2016;36(2):141‐148. 26846287 10.1007/s10875-016-0236-y

[4] Odat H , Alqudah M . Prevalence and pattern of humoral immunodeficiency in chronic refractory sinusitis. Eur Arch Oto‐Rhino‐Laryngol. 2016;273(10):3189‐3193. 10.1007/s00405-016-3981-x 26975445

[5] May A , Zielen S , von Ilberg C , Weber A . Immunoglobulin deficiency and determination of pneumococcal antibody titers in patients with therapy‐refractory recurrent rhinosinusitis. Eur Arch of Oto‐Rhino‐laryngol. 1999;256(9):445‐449. 10.1007/s004050050186 10552223

[6] De Gracia J , Rodrigo MJ , Morell F , et al. IgG subclass deficiencies associated with bronchiectasis. Am J Respir Crit Care Med. 1996;153(2):650‐655. 8564113 10.1164/ajrccm.153.2.8564113

[7] Carruthers MN , Khosroshahi A , Augustin T , Deshpande V , Stone JH . The diagnostic utility of serum IgG4 concentrations in IgG4‐related disease. Ann Rheum Dis. 2015;74(1):14‐18. 24651618 10.1136/annrheumdis-2013-204907

[8] Stone JH , Zen Y , Deshpande V . IgG4‐related disease. N Engl J Med. 2012;366(6):539‐551. 22316447 10.1056/NEJMra1104650

[9] Zen Y , Nakanuma Y . IgG4‐related disease: a cross‐sectional study of 114 cases. Am J Surg Pathol. 2010;34(12):1812‐1819. 21107087 10.1097/PAS.0b013e3181f7266b

[10] Picard C , Al‐Herz W , Bousfiha A , et al. Primary immunodeficiency diseases: an update on the Classification from the International Union of Immunological Societies Expert Committee for Primary Immunodeficiency 2015. J Clin Immunol. 2015;35(8):696‐726. 26482257 10.1007/s10875-015-0201-1 PMC4659841

[11] Varghese JL , Fung AWS , Mattman A , et al. Clinical utility of serum IgG4 measurement. Clin Chim Acta. 2020;506:228‐235. 32272158 10.1016/j.cca.2020.04.001

[12] Hegade VS , Sheridan MB , Huggett MT . Diagnosis and management of IgG4‐related disease. Frontline Gastroenterol. 2019;10(3):275‐283. 31288262 10.1136/flgastro-2018-101001 PMC6583577

[13] Hao M , Liu M , Fan G , Yang X , Li J . Diagnostic value of serum IgG4 for IgG4‐related disease: a PRISMA‐compliant systematic review and meta‐analysis. Medicine. 2016;95(21):e3785. 27227950 10.1097/MD.0000000000003785 PMC4902374

[14] Abraham M , Khosroshahi A . Diagnostic and treatment workup for IgG4‐related disease. Expert Rev Clin Immunol. 2017;13(9):867‐875. 28701054 10.1080/1744666X.2017.1354698 PMC5896560

[15] Inoue D , Yoshida K , Yoneda N , et al. IgG4‐related disease: dataset of 235 consecutive patients. Medicine. 2015;94(15):e680. 25881845 10.1097/MD.0000000000000680 PMC4602507

[16] Ma Y , Chen L , Xu Y , et al. Clinical and pathological features of patients with antineutrophil cytoplasmic antibody‐associated vasculitides concomitant with IgG4‐related disease. Int J Rheum Dis. 2019;22(12):2143‐2150. 31631507 10.1111/1756-185X.13726

[17] Huijbers MG , Querol LA , Niks EH , et al. The expanding field of IgG4‐mediated neurological autoimmune disorders. Eur J Neurol. 2015;22(8):1151‐1161. 26032110 10.1111/ene.12758

[18] Aksu G , Genel F , Koturoglu G , Kurugol Z , Kutukculer N . Serum immunoglobulin (IgG, IgM, IgA) and IgG subclass concentrations in healthy children: a study using nephelometric technique. The. Turk J Pediatr. 2006;48(1):19‐24. 16562781

[19] Grunewald O , Lopez B , Brabant S , et al. Immunoglobulin G (IgG) and IgG subclass reference intervals in children, using Optilite(R) reagents. Clin Chem Lab Med. 2018;56:1319‐1327. 29630506 10.1515/cclm-2018-0001

[20] Puissant‐Lubrano B , Peres M , Apoil PA , Congy‐Jolivet N , Roubinet F , Blancher A . Immunoglobulin IgA, IgD, IgG, IgM, and IgG subclass reference values in adults. Clin Chem Lab Med. 2015;53(12):e359‐e361. 25720098 10.1515/cclm-2014-1186

[21] Sarnago A , Pascual RM , Moreno MJ , Laiz B , Fuster O . IgG subclasses quantitation: analytical performance of The Binding Site SPAPLUS(R) human assay and comparison with Siemens BNII(R) assay. Clin Biochem. 2018;51:85‐89. 28912097 10.1016/j.clinbiochem.2017.09.004

[22] Michaut L , Laurent N , Kentsch K , Spindeldreher S , Deckert‐Salva F . Stability of anti‐immunotherapeutic antibodies in frozen human serum samples. Bioanalysis. 2014;6(10):1395‐1407. 24958123 10.4155/bio.14.97

[23] Sprogoe U , Yazer MH , Rasmussen MH , Antonsen B , Bistrup C , Assing K . Minimal variation in anti‐A and ‐B titers among healthy volunteers over time: Implications for the use of out‐of‐group blood components. The journal of trauma and acute care surgery. 2017;82(6S Suppl 1):S87‐s90. 28333830 10.1097/TA.0000000000001432

[24] Dansk Selskab for Klinisk Immunologi . Transfusionsmedicinske Standarder (Version 4.3). 2017. https://dski.dk/wp-content/uploads/2020/03/tms-5-1.pdf

[25] The Binding Site . SPA Plus—Assay Menu. 2020. https://www.bindingsite.com/en/our-products/clinical-chemistry/spaplus/assay-menu

[26] Schauer U , Stemberg F , Rieger CH , et al. IgG subclass concentrations in certified reference material 470 and reference values for children and adults determined with the binding site reagents. Clin Chem. 2003;49(11):1924‐1929. 14578325 10.1373/clinchem.2003.022350

[27] Carballo I , Alvela L , Pérez LF , et al. Serum concentrations of IgG4 in the Spanish adult population: relationship with age, gender, and atopy. PLOS One. 2016;11(2):e0149330. 26910567 10.1371/journal.pone.0149330 PMC4766298

[28] Santagostino A , Garbaccio G , Pistorio A , et al. An Italian national multicenter study for the definition of reference ranges for normal values of peripheral blood lymphocyte subsets in healthy adults. Haematologica. 1999;84(6):499‐504. 10366792

[29] Comans‐Bitter WM , de Groot R , van den Beemd R , et al. Immunophenotyping of blood lymphocytes in childhood. Reference values for lymphocyte subpopulations. J Pediatr. 1997;130(3):388‐393. 9063413 10.1016/s0022-3476(97)70200-2

[30] Aziz N , Detels R , Quint JJ , Gjertson D , Ryner T , Butch AW . Biological variation of immunological blood biomarkers in healthy individuals and quality goals for biomarker tests. BMC Immunol. 2019;20(1):33. 31521107 10.1186/s12865-019-0313-0 PMC6744707

